# Androgen Receptor-CaMKK2 Axis in Prostate Cancer and Bone Microenvironment

**DOI:** 10.3389/fendo.2018.00335

**Published:** 2018-06-18

**Authors:** Ushashi C. Dadwal, Eric S. Chang, Uma Sankar

**Affiliations:** Department of Anatomy and Cell Biology, Indiana University School of Medicine, Indianapolis, IN, United States

**Keywords:** castrate-resistant prostate cancer, androgen-deprivation therapy, CAMKK2, bone–tumor microenvironment, treatment induced bone loss

## Abstract

The skeletal system is of paramount importance in advanced stage prostate cancer (PCa) as it is the preferred site of metastasis. Complex mechanisms are employed sequentially by PCa cells to home to and colonize the bone. Bone-resident PCa cells then recruit osteoblasts (OBs), osteoclasts (OCs), and macrophages within the niche into entities that promote cancer cell growth and survival. Since PCa is heavily reliant on androgens for growth and survival, androgen-deprivation therapy (ADT) is the standard of care for advanced disease. Although it significantly improves survival rates, ADT detrimentally affects bone health and significantly increases the risk of fractures. Moreover, whereas the majority patients with advanced PCa respond favorably to androgen deprivation, most experience a relapse of the disease to a hormone-refractory form within 1–2 years of ADT. The tumor adapts to surviving under low testosterone conditions by selecting for mutations in the androgen receptor (AR) that constitutively activate it. Thus, AR signaling remains active in PCa cells and aids in its survival under low levels of circulating androgens and additionally allows the cancer cells to manipulate the bone microenvironment to fuel its growth. Hence, AR and its downstream effectors are attractive targets for therapeutic interventions against PCa. Ca^2+^/calmodulin-dependent protein kinase kinase 2 (CaMKK2), was recently identified as a key downstream target of AR in coordinating PCa cell growth, survival, and migration. Additionally, this multifunctional serine/threonine protein kinase is a critical mediator of bone remodeling and macrophage function, thus emerging as an attractive therapeutic target downstream of AR in controlling metastatic PCa and preventing ADT-induced bone loss. Here, we discuss the role played by AR-CaMKK2 signaling axis in PCa survival, metabolism, cell growth, and migration as well as the cell-intrinsic roles of CaMKK2 in OBs, OCs, and macrophages within the bone microenvironment.

## Introduction

Prostate cancer (PCa) is the second leading cause of cancer-related deaths in American men and accounts for 15% of all cancers diagnosed in men worldwide (1, 2). The American Cancer Society estimates that in 2018 alone, 164,690 men will be newly diagnosed with PCa and 29,430 men will die from it in the United States. Routine testing of prostate serum antigen (PSA) levels has resulted in early diagnosis and treatment of PCa. Consequently, men with early-stage PCa have a high, near 100%, 10-year rate of survival. Among patients with non-localized disease, however, about 40% develop metastases to distant sites such as bone, lymph nodes, lung, and brain and their 5-year survival rate drops dramatically to 30% (3). PCa displays a preferential tropism toward bone which is the primary site of metastasis in 80% of patients with advanced disease (4). Metastatic PCa becomes lodged in the bone marrow (BM)-rich axial skeleton, which provides the perfect “soil” for the disease to develop to an advanced form often termed “castrate-resistant PCa (CRPC)” as it is resistant to hormone-ablation.

Bone is an organ of utmost importance in PCa. Bone metastasis is often a leading cause of patient mortality in PCa (4). Once they “home” and colonize the bone, PCa cells disrupt the homeostatic balance between bone-forming osteoblasts (OBs) and bone-resorbing osteoclasts (OCs). Similar to breast cancer, PCa stimulates osteolysis. However, a unique feature of bone-lodged PCa cells is that they stimulate the OBs to produce weak woven bone instead of the strong lamellar bone that is normally synthesized. Such skeletal-related events (SREs) triggered by PCa in the bone culminate in pathological fractures, spinal cord compression, and sclerosis, detrimentally affecting the overall quality of life and survival rate among patients (5–8).

Prostate cancer cells express the androgen receptor (AR) and are heavily reliant on androgens for growth and survival. Hence, most patients with locally advanced or metastatic PCa receive androgen-deprivation therapy (ADT) as a gold standard treatment (9). Although it significantly improves survival rates, ADT detrimentally affects skeletal health, causing tremendous bone loss and rendering the patients at risk for fragility fractures (10). Therapies that inhibit bone resorption such as bisphosphonates prevent ADT-induced bone loss and may additionally delay bone colonization by the tumor by interfering with its ability to manipulate the bone microenvironment (11). PCa patients initially respond positively to ADT, though most experience a relapse of the cancer to a hormone-refractory form called CRPC, which occur when cancer cells adapt to growth under low androgen conditions by constitutively upregulating AR (12). Consequently, AR and its downstream effectors are attractive therapeutic targets to combat tumor growth in androgen-resistant PCa. Indeed, clinical studies indicate that AR inhibitors such as enzalutamide delay SREs and improve survival rates in patients (13–15). Still, novel therapies that preserve musculoskeletal heath while significantly inhibiting tumor growth are acutely needed in the clinic.

In this review, we will briefly discuss the steps involved in bone metastasis of PCa, the role of AR activation in the development of CRPC and skeletal complications associated with ADT. We will additionally discuss recent findings that identify Ca^2+^/calmodulin (CaM)-dependent protein kinase kinase 2 (CaMKK2), an AR-regulated gene with additional roles in bone remodeling and inflammation, as a novel therapeutic target to inhibit PCa growth and prevention of ADT-associated bone loss.

## Bone Metastasis of PCa

Prostate cancer cells show an almost exclusive tropism for bone. Although the exact mechanisms that drive bone metastasis are unknown, it has been proposed that the BM microenvironment may provide the ideal condition for the PCa cells to thrive. The “seed and soil” hypothesis proposed by Steven Paget in 1889, wherein the “seeds” or tumor cells develop a tropism and metastasize to the “soil” or target organ that is well suited or “fertile ground” for its growth (16) still remains a guiding principle in understanding the role BM microenvironment plays in bone metastasis of PCa.

Metastasis of PCa to bone involves several steps including decreased cell adhesion, epithelial to mesenchymal transition (EMT), local migration, invasion, intravasation into the circulation, homing, and colonization of bone (17, 18). Cell–cell adhesion in normal prostate epithelium is maintained by integrins and tight junctions composed of cell adhesion molecules, such as selectins and cadherins. There are two main types of cadherins, E-cadherin and N-cadherin, expressed by epithelial cells and mesenchymal cells, respectively. During early transformation, prostate epithelial cells exhibit alterations in cell adhesion factors, including a downregulation of E-cadherin and an upregulation of N-cadherin, a process termed cadherin switching and a main feature in EMT. Decreased expression of integrins and Wnt-target protein β-catenin also play important roles in EMT (17–19). The next step is migration and it involves an upregulation of focal adhesion. During normal cell migration, focal adhesions formed on the leading edges of the cells are used as anchors by the cells to pull themselves forward over the extracellular matrix (ECM). Disassembly of focal adhesions on the rear edge of the cell enables the cell to move forward (20). This process involves the binding of focal adhesion kinases (FAKs) to integrins and their subsequent activation by the Src family of kinases, initiating signaling events including those involving Rho that regulate focal adhesion turnover and migration. Expression of FAK and Src as well as Rho activities are elevated in bone metastases and CRPC, indicating increased focal adhesion turnover and cell mobility.

Once the transformed prostate epithelial cells gain the ability to migrate, they need to dissociate from the ECM, which is composed of the basement membrane and connective tissue. Prostate epithelial cells that have undergone mesenchymal transition have the ability to secrete proteases such as matrix metalloproteases and serine proteinases, such as urokinase-type plasminogen activator and PSA, which partially degrade the ECM, allowing the cells to disseminate, invade the surrounding tissue and intravasate into blood vessels (17, 20, 21). Homing to the target organ is only possible if the PCa cells survive in the circulation, and they achieve this by attaching to the vascular endothelium. PCa cells have been shown to interact with BM endothelial cells (BMECs) with high affinity through a mechanism involving E-selectin receptor on PCa cells and E-selectin on BMECs and integrins such as αVβ3, αVβ1, and α3β1 (18). Additionally, CD44 on PCa cells binds to vascular cell adhesion molecule 1 on BMECs in a process that is enhanced by IL-17 and IGF1 in circulation. The subsequent homing of PCa cells to bone is facilitated by multiple chemokine-mediated mechanisms. For instance, BM stromal cells and OBs in the bone secrete C–X–C motif chemokine ligand 12 (CXCL12) or stromal derived factor-1 (SDF1) whereas PCa cells express its receptor CXCR4. CXCL12/SDF–CXCR4 interaction allows PCa cells to home to the bone, adapting a similar mechanism as the one utilized by hematopoietic stem cells (22). Additionally, CXCL12/SDF1 from OBs activates the expression of the adhesion molecule αVβ3 integrin on PCa cells that further contribute to their homing to the BM. Further, the expression of yet another chemokine ligand CXCL16 allows PCa cells to recruit and convert CXCR6-expressing mesenchymal stem cells into cancer-associated fibroblasts that also secrete high levels of CXCL12/SDF1. Finally, recent reports provide evidence for microRNA (miR)-containing exosomes from PCa cells arriving early in the BM to enable the modification of the bone microenvironment to favor cancer cell homing to the bone (23–25).

Colonization of the bone by PCa is aided by their ability to (a) attach to the bone matrix and (b) manipulate the BM microenvironment into favoring their growth and survival. PCa express two key integrins αVβ3 and α2β1, which allow the cells to attach to the bone matrix and migrate along the surface to identify suitable “niches” for their outgrowth. PCa cells preferentially home to OB-rich niches within the bone, allowing physical contact between these two cell types, facilitated in part by adhesion molecules such as cadherin-11 expressed on both OBs and malignant PCa cells (26, 27). Interestingly, physical contact between PCa and OBs appear to disrupt the normal order of matters within the bone. Kimura et al. noted that in the presence of PCa cells, the bone-resident OBs which usually line neatly along the collagen matrix become disorganized and that this anisotropy requires cell–cell contact (28). Unlike other solid tumor malignancies which are mostly osteolytic, bone-metastatic PCa is primarily an osteoblastic disease driven in part by the ability of PCa cells to perform “osteomimicry” wherein they adopt the genetic and phenotypic characteristics of OBs (29). OB growth and differentiation are governed by complex signaling pathways, such as Wnts, bone morphogenic proteins (BMPs), insulin growth factor (IGF), and transforming growth factor β (TGF-β) (30, 31). In contrast, OC differentiation is regulated by receptor activator of NF-κB ligand (RANKL), osteoprotegerin, parathyroid hormone, and TGF-β. Differentiated OBs secrete these factors, but many are also released from the bone matrix by OCs themselves during bone resorption. Interestingly, bone-lodged PCa cells produce many of the same factors that stimulate the proliferation and differentiation of OBs and OCs (17, 30). In addition to producing factors that favor bone cell differentiation, PCa cells also induce other cell types to transdifferentiate into OBs (32). Recently, Lin et al. reported an endothelial cell-to-OB conversion as one of the mechanisms underlying osteoblastic bone disease in PCa (33). These authors showed that PCa induces the overexpression of BMP4 in BMECs driving their transdifferentiation to OBs (33). Recent studies from multiple myeloma highlight the importance of osteocytes, the most abundant bone cells, in tumor-bone interactions (34). Although studies have indicated a role for osteocytes in PCa (35), more research is needed to fully comprehend the contribution of these cells to bone metastasis by PCa. Taken together, these studies suggest that cancer cells disrupt the homeostatic mechanisms within the BM and hijack the normal paracrine and autocrine mechanisms regulating normal bone remodeling to create a “vicious cycle” that ultimately favors PCa colonization and growth within the bone (Table 1).

**Table 1:** Growth factors involved in aiding skeletal metastasis of prostate cancer.

Factor	Role	Function	Source cells
CXCL12/SDF1	Homing	Binding partner to CXCR4	Osteoblasts (OBs) (36)

CXCR4	Homing	Binding partner to CXCL12	Tumor Cells (36)

E-selectin ligands	Colonizing	Critical for initial tethering and rolling on E-selectin	Tumor Cells (37)

CXCR6	Progression	Recruits and converts mesenchymal stem cells (MSCs) into Cancer-associated fibroblasts	MSCs (38)

BMP4	Progression	Drives endothelial cell conversion into OBs	Tumor cells (33)

IGF1	Progression	Stimulates proliferation of human prostate epithelial cells	Tumor cells (39)

Endothelin 1	Progression	Suppresses Dickkoph 1, increases OB mitogensis and osteoclast apoptosis	Tumor cells (40)

B7-H ligand	Progression	Evading immune cell surveillance	Tumor cells (41)

Androgens	Proliferation	Stimulate androgen receptor signaling mediated bone formation in OBs	Tumor cells (42)

## Androgens, AR, Bone, and ADT

Since the original description by Charles Huggins in 1942 of the heavy dependence of PCa on androgens and the benefits of orchiectomy in PCa patients, androgens, and AR have remained the main therapeutic targets in PCa treatment (43–46). In men, Leydig cells of the testis produce about 90% of the circulating androgens or testosterone and the adrenal cortex produces the remaining 10% (47). Testosterone diffuses into the prostate epithelial cells where it is converted into dihydrotestosterone (DHT) by the enzyme 5α-reductase (47, 48). DHT binds to AR, a member of the nuclear hormone receptor family of transcription factors. Upon ligand binding, AR translocates to the nucleus, undergoes homodimerization and binds to androgen response elements (ARE) within the promoters of AR-target genes such as PSA. AR then recruits cofactors and initiates the transcription of target genes that regulate proliferation, metabolism, and survival of PCa cells (45, 49, 50).

The goal of ADT is to starve the tumor cells of androgens by drastically diminishing their circulating amount (<5% of normal range). This is achieved by blocking testosterone production surgically *via* castration or chemically by treating patients with luteinizing hormone releasing hormone agonists or first generation antiandrogen drugs, such as flutamide, nilutamide, and bicalutamide, that competitively block DHT binding to AR (51). Testosterone is converted into estradiol, the primary male estrogen *via* aromatization and it binds to the estrogen receptor α (ERα) present on both OBs and OCs. OBs express both AR and ERα, whereas OCs express only ERα. These receptors promote OB survival, numbers, and activity, while ERα inhibits OC differentiation. Moreover, the combined action of these two nuclear receptors stimulate periosteal apposition and lengthening of the epiphyseal growth plate in men while maintaining their cortical and trabecular bone. The continued periosteal growth during adult life in men partially offsets age-related increase in endosteal bone loss (7, 10). All these processes are affected by ADT as it suppresses not only androgens but also estradiol, resulting in the abrogation of the stimulatory effect of androgens on OBs and the inhibitory effect of estradiol on OCs. This triggers increased bone turnover in patients on ADT, resulting in a significantly high rate of bone loss at 4.6% per year, which exceeds the annual bone loss in aging men and postmenopausal women (10). The maximum bone loss occurs during the first year of therapy, ranging from 1.5 to 4%, depending on the skeletal location examined (10). Thus, ADT renders these men, who are often older and possess lower bone mass to begin with, four times more likely to develop osteoporosis. This enhances their risk of fragility fractures and in turn, their mortality risk (7).

Nitrogen-containing bisphosphonates, such as alendronate, risedronate, and zoledronic acid, as well as denosumab, a monoclonal antibody to RANKL are all FDA-approved to treat osteoporosis in PCa patients on ADT. Selective ER modulators such as raloxifene and Toremifene have also been shown to preserve bone in clinical trials with PCa patients undergoing ADT (7, 15, 52–56). Moreover, second-generation antiandrogens, such as abiraterone and enzalutamide, as well as radiotherapies such as Radium-223 have shown to suppress tumor growth and delay SREs (13, 14, 57–60). Teriparatide, though FDA-approved, is not recommended for PCa patients at risk for bone metastasis. A list of current therapies and novel compounds in clinical trials in the treatment of bone-metastatic PCa are detailed in Tables 2 and 3.

**Table 2:** Androgen receptor (AR) targeted therapies—FDA-approved drugs in clinic.

Drug	Target	Mechanism of action	Clinical use	Reference
Abiraterone acetate	Cytochrome P450 c17 (CYP17)	Inhibits androgen biosynthesis	Castration-resistant and high-risk castration sensitive prostate cancer (PCa)	(57)

Enzalutamide (Xtandi)	AR	Inhibits nuclear translocation of the AR	Metastasized castrate-resistant prostate cancer	(14)

Leuprolide acetate	Luteinizing hormone releasing hormone	Inhibits secretion of luteinizing hormone, androgen, and estradiol	Approved for palliative treatment of advanced PCa	(61)

R-Bicalutamide (CASODEX)	Cytosolic AR	Inhibits androgen activity by binding cytosolic ARs and stimulating AR nuclear translocation	Approved for metastasized PCa	(62)

**Table 3:** Novel therapies against castrate-resistant prostate cancer (CRPC) currently in trials.

Drug	Target	Mechanism of action	Trial Status	Reference
**Novel androgen receptor (AR) therapeutics currently in clinical trial**				
ARN-509 (apalutamide)	Androgen receptor (AR)	Competitively inhibits transcription	Phase II	(57, 63, 64)
EPI-506	AR	Inhibits transcription	Phase II	(53)
AZD3514	AR	Inhibition of AR nuclear translocation and AR-regulating gene transcription	Phase I	(65)
Ketoconazole	Cytochrome P450 c17 (CYP17)	Inhibits adrenal testosterone synthesis	Phase II	(66)
MDV3100	AR	Inhibits AR binding and nuclear translocation of the AR	Phase I	(35)

**Other novel drug targets**				
Radium-223 (Xofigo)	Bone mineral hydroxyapatite	Induces double-strand DNA breaks	FDA approved for CRPC, bone metastasis	(67)
LGK974	Porcupine [PORCN] (WNT-specific acyltransferase)	Inhibits Wnt signaling	Phase I	(68)
Cytarabine (Cytosine Arabinoside)	DNA polymerase	Inhibits DNA synthesis	Phase II	(69)
Ipatasertib	AKT (protein Kinase B)	Inhibits three isoforms of AKT	Phase II	(55, 70)

## AR Activation in CRPC

Androgen-deprivation therapy results in diminished tumor burden in about 90% of patients with advanced PCa. However, over time, the cancer cells adapt undergo selection to proliferate and survive under low levels of circulating androgens by upregulating AR and becoming unresponsive to ADT. The disease at this stage is termed CRPC (17, 71). AR is the main driver of CPRC development, while a minority of metastatic PCa are associated with the loss of *p53, PTEN*, or *Rb* (13, 72). The main mechanisms for AR reactivation in CRPC include amplification leading to overexpression, activating mutations, structural gene alterations, expression of constitutively active variants, mutations in the AR that confer broader ligand specificity to the receptor, upregulation of co-regulators, increased expression of steroidogenic enzymes, as well as upregulation of cross-talk signal transduction pathways such as interleukin 6, STAT3, Src, and IGF that can activate AR in a ligand-independent manner (71). These mechanisms have been extensively reviewed elsewhere (21, 72–74). Gain-of-function AR splicing variants (AR-Vs) often lack portions of the ligand-binding domain (LBD) but possess constitutive transcriptional activity even in the absence of androgens. The most well-characterized among these is AR-V7 whose expression has been shown to increase in response to ADT and has been shown to confer resistance to drugs, such as abiraterone and enzalutamide that either block androgen synthesis or antagonize AR (75). AR-V7 was identified as the most frequently occurring variant in patients with CRPC and its expression correlates with increased disease recurrence (76–78). AR-V7 expression is associated with the upregulation of some AR-target genes relevant for proliferation and survival, such as *UBE2C, CCNA2, C-MYC, AKT1, EDN2*, and *ETS2* (71, 78).

Mechanisms that enable CRPCs to activate AR and continually acquire resistance to therapies underscore the importance of gaining a comprehensive understanding of the downstream effectors of AR signaling that play crucial roles in cancer progression, as they could serve as druggable targets in the treatment of CRPC. Several studies have attempted to identify AR-regulated genes by focusing on genome-wide AR-binding sites on cell lines and clinical samples, or by examining temporal regulation of androgen stimulation in one or more PCa cell lines such as LNCaP (harbors an LBD mutation of AR), VCaP (contains AR gene amplification) or C4-2B (a CRPC cell line) (66, 79–85). These studies have identified several AR-target genes with functions in gene transcription (*NKX3.1, FOX* family), growth stimulation (IGF1R), cell cycle regulation (*CDK6, UBE2C*), signaling (*MEK5, FKBP5*), autophagy (*ATG4B, ULK1, TFEB*), non-coding RNA (*miR-21, miR141*), glycolysis (*GLUT1*), and central metabolism [*MTOR*, Ca^2+^/CaM-dependent protein kinase kinase 2 (*CaMKK2*)]. Among these, *CaMKK2* has emerged as an attractive therapeutic candidate in PCa as it is a direct target of AR, containing AREs on its promoter and is consistently overexpressed in clinical CRPC samples as well as AR-positive PCa cell lines (86, 87).

## CaMKK2: A Molecular Hub Directed by AR in PCa Cells

Intracellular Ca^2+^ is a universal second messenger that regulates diverse cellular processes. Transient variations in intracellular Ca^2+^ are immediately sensed by the ubiquitous high-affinity intracellular Ca^2+^ receptor, CaM. This initiates a cascade of Ca^2+^/CaM-mediated signaling events that culminate in changes to key cellular events such as proliferation, differentiation, survival, and metabolism (88). In particular, Ca^2+^/CaM complexes bind to and activate CaM kinases (CaMKs), which are a family of multifunctional Ser/Thr protein kinases that includes CaMKK1, CaMKK2, CaMKI, CaMKII, and CaMKIV. The upstream kinases, CaMKKs 1 and 2, are activated through Ca^2+^/CaM binding and intramolecular phosphorylation. Binding of Ca^2+^/CaM allows the activation loop in CaMKs to unravel and expose a critical threonine residue that becomes phosphorylated by the two upstream CaMKKs, resulting in their full activation, triggering the formation of a CaMK signaling cascade that is regulated by Ca^2+^/CaM at multiple levels (89–91). Interestingly, unlike CaMKK1, which is solely dependent on Ca^2+^/CaM for activity, CaMKK2 possesses considerable autonomous activity in the absence of Ca^2+^/CaM. This autonomous activity is regulated by phosphorylation by Ca^2+^/CaM-independent kinases such as glycogen synthase kinase 3β (GSK3β) and cyclin-dependent kinase 5 (CDK5) (92, 93). As it is not dependent on rapid fluxes in intracellular Ca^2+^ for basal activity, CaMKK2 is capable of responding to other stimuli of longer duration and phosphorylating novel substrates outside of the CaMK cascade. Indeed, CaMKK2 (not CaMKK1) directly phosphorylates and activates adenosine monophosphate activated protein kinase (AMPK), a heterotrimeric kinase that co-ordinates cellular energy balance, autophagy, cell proliferation, and cytoskeletal organization (94, 95). The CaMKK2–AMPK pathway plays key roles in the regulation of hypothalamic feeding behavior, hepatic gluconeogenesis, adipocyte differentiation, and macroautophagy (94, 96–98). Recent studies indicate roles for CaMKK2 in hepatic cancer, macrophage-mediated inflammation, and bone remodeling through non-AMPK-mediated mechanisms (99–103).

CaMKK2 is increasingly being considered a hub of signaling mechanisms that regulate PCa cell metabolism, proliferation and migration downstream of AR (104). Frigo et al. identified the presence of an AR-binding region located 2.3-kb upstream of the *CaMKK2* transcriptional start site and reported the recruitment of AR to this region in an androgen-dependent manner (87). These authors also found that the knockdown of *CaMKK2* or its pharmacological inhibition using a selective inhibitor STO-609 or inhibition of the CaMKK2-target protein AMPK abrogates PCa cell migration and invasion (68, 87, 105). Overexpression of CaMKK2 alone was sufficient to induce AMPK phosphorylation and facilitate PCa cell migration, implying that androgens promote PCa cell migration through an AR-CaMKK2-AMPK signaling axis (87). Massie et al. integrated genome-wide AR-binding transcript profiling with an analysis of androgen-stimulated recruitment of the transcriptional machinery to a core set of AR-binding sites and identified *CaMKK2* to be consistently enriched in PCa clinical cohorts, in a pattern similar to that of the established PCa marker AMACR (86). Similar to previous reports (87), these authors also observed AR recruitment to *CaMKK2* promoter in both androgen-dependent and CRPC cell lines and an early upregulation of the CaMKK2 transcripts and protein within 4 and 12 h of androgen stimulation, respectively, indicating direct AR regulation (86). Subsequent functional studies identified CaMKK2 as a key effector of AR in stimulating glycolysis through its activation of AMPK and phosphofructokinase (PFK), which in turn drives anabolism and PCa cell proliferation (86). Of note, the AR-CaMKK2–AMPK–PFK axis does not affect cellular biosynthesis through mTOR in PCa, indicating its primary role in regulating glucose uptake and lactate production. *In vivo* inhibition of CaMKK2 using STO-609 resulted in a significant reduction in the growth of C4-2B xenografts in nude mice, and this treatment was additive with AR inhibition achieved *via* castration (86). It should be noted that CaMKK2 inhibition by itself did not affect the size of the normal prostate or its epithelium in nude mice, and the *CaMKK2^−/−^* mice do not possess any prostate anomalies or fertility deficits (86, 87). Thus, the inhibition of CaMKK2, rather than AR itself, may offer greater selective advantage over PCa at all stages.

Karacosta et al. examined PCa in clinical samples and found strong CaMKK2 immunoreactivity in the epithelium of malignant glands, compared to extremely low expression in the adjacent normal epithelium (106). Moreover, CaMKK2 staining intensity increased with the Gleason score of the tumors, and the staining pattern shifted from predominantly cytoplasmic to perinuclear and nuclear (106). CaMKK2 intensity increased with tumor progression in a transgenic adenocarcinoma of the mouse prostate (TRAMP) mouse model of PCa, and its expression was higher in castration-resistant tumor xenografts than androgen-responsive ones. These authors also observed upregulation of CaMKK2 as well as its nuclear translocation in LNCaP following DHT treatment and a reversal with androgen withdrawal. Further, silencing of *CaMKK2* using small interfering RNA elicited G1 arrest of LNCaP cells, reducing their proliferation, along with lowering the levels of PSA as well as AR-regulated cell cycle proteins such as cyclin D1 and hyperphosphorylated Rb (106). Karacosta et al. proposed a novel positive feedback loop in the PCa in which CaMKK2 is induced by AR, and it in turn stabilizes AR to promote its transcriptional activity and cell cycle progression (106). In a recent follow-up study, these authors confirmed the higher nuclear expression of CaMKK2 in CRPC C4-2 cells, and showed that this occurs due to the association of CaMKK2 with nuclear pore complexes through its direct interaction with nucleoporin 62 (NUP62) (107). These authors showed that silencing NUP62 reduces the growth and viability of C4-2 cells, and provided evidence for the recruitment of NUP62, CaMKK2, and AR complexes to the AR-binding regions in the promoters of target genes such as *PSA*, suggesting a novel CaMKK2-NUP62 mechanism of AR transcriptional regulation in advanced PCa (107).

Similar to the aforementioned studies, Shima et al. performed genome-wide analysis of a small set of clinical samples and found a sixfold higher *CaMKK2* expression in PCa compared to normal prostate (108). However, in contrast to previous studies (82, 87, 106, 107), these authors provide evidence for an inhibitory role for CaMKK2 to AR signaling and hypothesize that while CaMKK2 supports growth of tumors in early PCa, it inhibits excessive proliferation in CRPC (108). Whereas additional studies are warranted to validate these intriguing findings and hypotheses, the consensus emerging from all of these studies is that CaMKK2 is a key effector of AR signaling in PCa cells, regulating cell cycle by stabilizing AR, cell migration through AMPK signaling, and glycolysis by activating the AMPK–PFK pathway. AR is essential for PCa cell viability, proliferation, invasion, and bone metastasis, and the tumor cells are under constant selective pressure to maintain AR signaling, especially under the conditions of low testosterone such as ADT (86). Therefore, targeting downstream effectors such as CaMKK2 would be an effective approach to abrogate AR signaling in metastatic PCa.

## CaMKK2 in Bone Microenvironment

### CaMKK2 and Bone Cells

Prostate cancer recruits OBs and OCs within the bone microenvironment and transforms them into entities that support tumor growth (30). Studies discussed above show that CaMKK2 is expressed in PCa cells where it acts as a molecular hub downstream of AR in regulating tumor cell growth. CaMKK2 is expressed by OBs and OCs and plays important cell-intrinsic roles in these cells (99). During homeostatic conditions, CaMKK2 stimulates OC differentiation by activating phosphorylated form of cyclic adenosine monophosphate (cAMP) response element binding protein (pCREB) and its transcriptional target, nuclear factor of activated T cells c1 in a CaMKIV-dependent manner. Hence, inhibition or deletion of CaMKK2 inhibits OCs. On the other hand, CaMKK2 inhibits OB differentiation by inhibiting cAMP-protein kinase A (PKA) signaling under normal conditions. Therefore, the inhibition or absence of CaMKK2 relieves this inhibition and results in the stimulation of OB differentiation (99). Mice null for *CaMKK2* possess higher bone mass along with significantly more OBs and fewer multinuclear OCs. Inhibition of CaMKK2 promotes bone fracture healing, and confers protection from ovariectomy and age-related osteoporosis (99, 100, 102). Taken together, these studies reveal profound roles for CaMKK2 in the two main bone cell types that interact with PCa in the bone microenvironment.

### CaMKK2 and Macrophages

**Figure 1:**
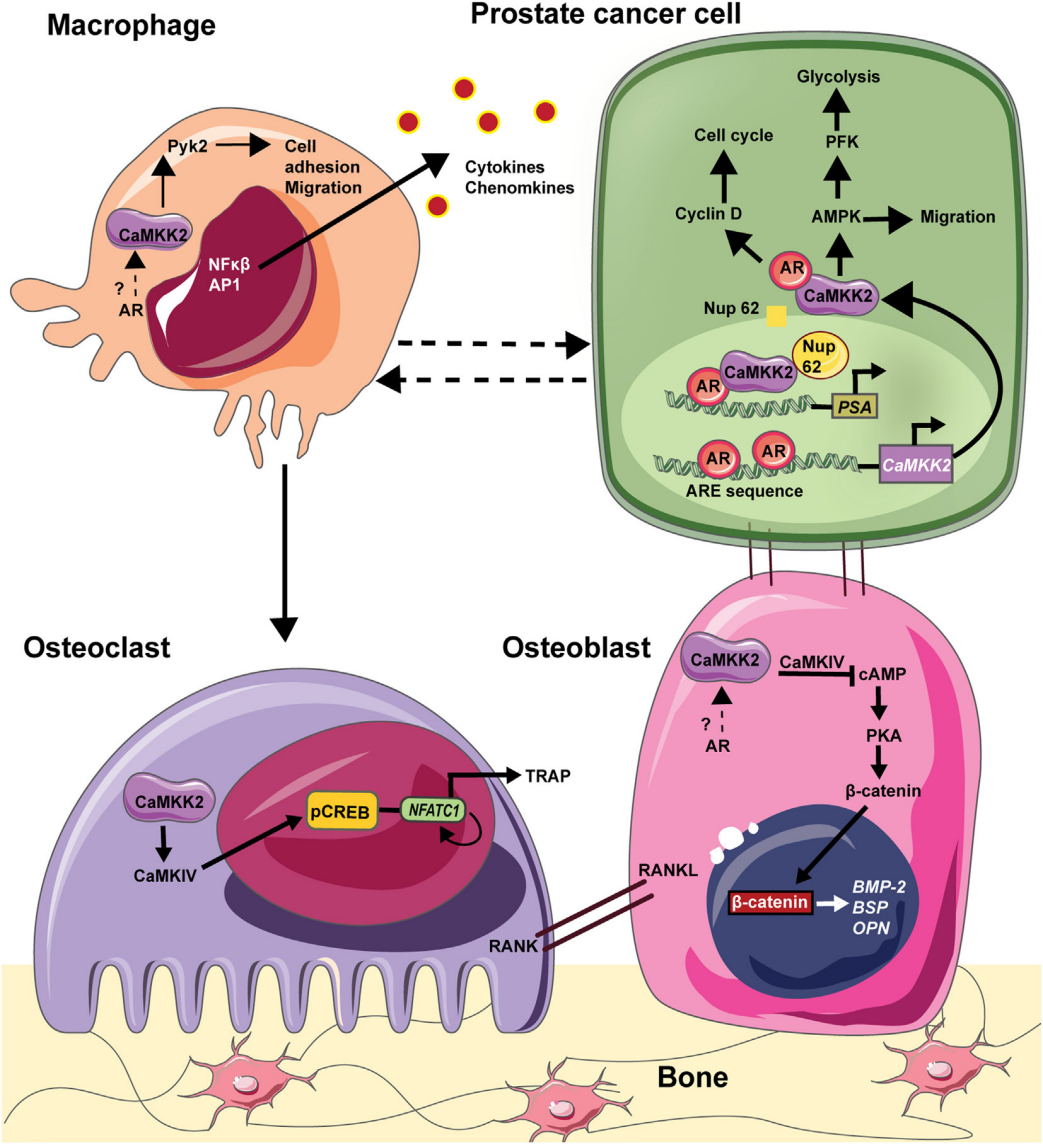
CAMKK2 as a molecular hub downstream in the bone–prostate cancer (PCa) microenvironment. In PCa cells, the androgen receptor (AR) binds to androgen response element (ARE) on *CaMKK2* promoter which is situated upstream of the transcriptional start site. Thus, CaMKK2 is a direct transcriptional target of AR and its expression is highly elevated in metastatic PCa. Once transcribed and translated, CaMKK2 binds to AR initiating a positive feedback loop to stimulate AR transcriptional activity in the activation of AR-dependent genes that regulate cell cycle progression such as cyclin D. Additionally, CaMKK2 through its activation of AMPK regulates PCa cell migration. CaMKK2-AMPK signaling pathway also regulates cellular glycolysis *via* the activation of phosphofructokinase (PFK). This drives PCa cell anabolism and in turn promotes cell proliferation and tumor growth. Furthermore, in CRPCs, CaMKK2 binds to nucleoporin 62 (NUP62) to enter the nucleus, where it along with AR and NUP62 are recruited to the ARE in the promoters of downstream targets such as prostate serum antigen (PSA). PCa cells that metastasize to the bone physically interacts with OBs to alter their organization and function. Although both AR and CaMKK2 are expressed in OBs, whether CaMKK2 operates downstream of AR in these cells is not known. In OBs, CaMKK2 signaling inhibits cyclic adenosine monophosphate (cAMP) production and protein kinase A (PKA) activation. PKA is an important regulator of OB differentiation. Thus, the inhibition of CaMKK2 would relieve this inhibition of PKA signaling and OB differentiation. In osteoclasts (OCs), CaMKK2 signaling through CaMKIV-pCREB activates nuclear factor of activated T cells c1 (NFATc1), which is the master regulator of OC differentiation. In macrophages, CaMKK2 regulates cytoskeletal rearrangement *via* its regulation of Pyk2. Moreover, CaMKK2-CaMK1 signaling regulates cytokine/chemokine production by macrophages. Thus, CaMKK2 is a key component of AR signaling in PCa cells and additionally regulates multiple cell types that constitute the tumor microenvironment within the bone.

Immune cells, such as macrophages and lymphocytes, are also part of the bone–tumor microenvironment and play important roles in tumor growth and bone metastasis (109). For example, chronic inflammation sustained by macrophage activation plays a pivotal role in the regulation of tumor microenvironment in many solid tumors (104). Chronic inflammatory conditions existing within the tumor recruit myeloid cells and induce their differentiation into tumor-associated macrophages, the infiltration of which negatively correlates with prognosis in advanced PCa. Recently, Roca et al. reported that macrophage-driven efferocytosis accelerates CXCL5-mediated inflammation and PCa growth within the bone (110). Among immune cells, CaMKK2 is selectively expressed in macrophages and its ablation impairs their ability to spread, phagocytose, and produce inflammatory cytokines and chemokines in response to lipopolysaccharides (101). CaMKK2 regulates metabolic responses and cytokine release in response toll-like receptor/integrin stimulation in macrophages. Indeed, *Camkk2^−/−^* mice are resistant to irritants that lead to systemic inflammation (101). Thus, CaMKK2 plays roles in multiple cell types, including OBs, OCs, and macrophages, that form the PCa microenvironment in the bone. AR is expressed in OBs and macrophages, and it plays an indirect role in OCs through ERα. However, whether CaMKK2 plays a role downstream of AR in OBs and macrophages is unknown. Nevertheless, we hypothesize that AR signaling in PCa cells uses CaMKK2 as a downstream hub regulating several molecular mechanisms in OBs, OCs, and macrophages to manipulate the BM niche to the benefit of the cancer cells (Figure 1).

## Perspectives and Concluding Remarks

Complex mechanisms employed by PCa cells allow it to home and thrive in bone, their preferred site of metastasis. Once lodged in the bone, the cancer cells recruit OBs, OCs, and macrophages within the skeletal niche to become entities that secrete growth factors and chemokines that allow the PCa cells to proliferate and survive even under low circulating testosterone conditions such as following ADT. AR signaling remains critical for PCa cell survival even under ADT and this creates selective pressure for the generation of *AR* gene mutations that facilitate the constitutive activation of the AR signaling cascade. Thus, AR and its downstream effectors are attractive therapeutic targets against bone-metastatic PCa.

The CaMKK2-AMPK signaling pathway operates downstream of AR to mediate PCa cell cycle, metabolism, migration, and invasion. CaMKK2 inhibition interferes with the growth and survival of bone-lodged PCa, and will presumably interfere with its ability to secrete factors that modify OBs into cancer-promoting entities. Similar to PCa, AR signaling plays an active pro-survival role in OBs. However, whether it operates upstream of CaMKK2 in OBs is unclear. Various signaling pathways, including cAMP-PKA, CDK5, and GSK3, have been implicated as upstream regulators of CaMKK2 in other cell types. In addition to AR-binding elements, *CaMKK2* promoter also contains consensus-binding sites for several transcription factors including runt-related transcription factor 2 (RUNX2), the master regulator of OB differentiation. In macrophages, CaMKK2 is activated by toll-like receptors, G_q_-coupled receptors, and voltage-gated Ca^2+^ channels on the plasma membrane (101). Although monocytes express AR, its role in the regulation of CaMKK2 in these cells is unclear. Nevertheless, we can conclude from the studies discussed above that the AR-CaMKK2 signaling axis acts as a molecular hub promoting PCa survival and in turn its ability to manipulate the bone microenvironment. Cell-intrinsic roles of CaMKK2 in OBs, OCs, and macrophages may aid in this process, ultimately enhancing the malignancy, SREs, and bone fragility.

In addition to the studies reviewed herein, CaMKK2 inhibition or genetic ablation has been shown to protect against diet-induced glucose intolerance, insulin resistance, diabetes, hepatocellular carcinoma, and non-alcoholic high fat liver disease [reviewed in Ref. (111)]. In case of PCa, CaMKK2 emerges as an attractive and druggable target downstream of AR that when inhibited, abrogates tumor growth, inhibits macrophage-mediated inflammation, and improves bone health. Future studies will provide a comprehensive understanding of the precise molecular mechanisms by which CaMKK2 regulates PCa cells as well as how AR-CaMKK2 signaling in these cells affects CaMKK2 function in bone cells and macrophages that constitute the bone microenvironment. Nonetheless, highly selective small molecule inhibitors of CaMKK2 should be developed as potent “dual-hit” therapeutic interventions to abrogate bone-metastatic PCa growth while preventing ADT-associated bone loss. Together with improving bone mass and strength in PCa patients, who are often elderly, CaMKK2 inhibition would offer the best odds for long-term disease-free survival.

## Author Contributions

UD and US contributed to conception, design, and writing of the manuscript. EC contributed to the literature search and drafted tables for manuscript. US critically reviewed the drafts of the manuscript and wrote the final version. UD and US read and approved the final manuscript.

## Conflict of Interest Statement

The authors declare that the research was conducted in the absence of any commercial or financial relationships that could be construed as a potential conflict of interest.
